# An engineered Fc fusion protein that targets antigen-specific T cells and autoantibodies mitigates autoimmune disease

**DOI:** 10.1186/s12974-023-02974-9

**Published:** 2023-12-06

**Authors:** Mathangi Janakiraman, Alexei Leliavski, Jeeva Varadarajulu, Dieter Jenne, Gurumoorthy Krishnamoorthy

**Affiliations:** 1 Research Group Neuroinflammation and Mucosal Immunology, Max Planck Institute of Biochemistry, Martinsried, Germany; 2 Max Planck Institute of Neurobiology, Martinsried, Germany

**Keywords:** Multiple sclerosis, EAE, Fc fusion, T cells, Autoantibodies, Tolerance, Myelin oligodendrocyte glycoprotein (MOG)

## Abstract

Current effective therapies for autoimmune diseases rely on systemic immunomodulation that broadly affects all T and/or B cell responses. An ideal therapeutic approach would combine autoantigen-specific targeting of both T and B cell effector functions, including efficient removal of pathogenic autoantibodies. Albeit multiple strategies to induce T cell tolerance in an autoantigen-specific manner have been proposed, therapeutic removal of autoantibodies remains a significant challenge. Here, we devised an approach to target both autoantigen-specific T cells and autoantibodies by producing a central nervous system (CNS) autoantigen myelin oligodendrocyte glycoprotein (MOG)-Fc fusion protein. We demonstrate that MOG-Fc fusion protein has significantly higher bioavailability than monomeric MOG and is efficient in clearing anti-MOG autoantibodies from circulation. We also show that MOG-Fc promotes T cell tolerance and protects mice from MOG-induced autoimmune encephalomyelitis. This multipronged targeting approach may be therapeutically advantageous in the treatment of autoimmunity.

**Supplementary Information:**

The online version contains supplementary material available at 10.1186/s12974-023-02974-9.

## Introduction

Active collaboration of antigen-specific T and B cells is a common feature of many autoimmune diseases such as multiple sclerosis (MS), an inflammatory disorder of the central nervous system (CNS) [1–3]. Such interactions lead to undesirable sequelae of activation of autoreactive T cells, B cells, and the production of autoantibodies which together contribute to autoimmune pathology [3]. Despite the appreciation of the importance of antigen-specific T cell/B cell cooperation in autoimmune pathogenesis, many currently available treatments for autoimmune diseases rely on global targeting of either T cells and/or B cells leading to broader immune suppression [4]. The development of therapeutic strategies to specifically blunt antigen-specific immune responses remains an important unresolved challenge.

The antigen-specific therapeutic approaches that have been experimentally evaluated have predominantly utilized fragments of autoantigens to induce T cell tolerance [5]. The approaches to deliver T cell antigenic epitopes range from the targeted expression of cognate antigen on antigen-presenting cells [6–8] to the use of nanoparticles as antigen delivery systems to ensure the gradual release of antigen over a long duration [9, 10], the use of synthetic proteins with antigenic epitopes [11], and DNA [12] or RNA vaccines [13]. Using these approaches, it is well established that antigen-specific T cells can be tolerized through the induction of anergy, clonal deletion, and induction of regulatory cells, etc., to mitigate autoimmune disease. While these approaches have shown efficacy in short term, their long-term effects are yet to be determined. These approaches, however, fail to target B cell compartment especially the autoantibodies which are crucial for the exacerbation of autoimmune pathology. The design of antigen-specific autoantibody targeting agents pose several challenges. First, many pathogenic antibodies are known to target conformational epitopes which require properly folded antigen. Second, these autoantibodies have a wide range of affinities and are typically present at low titers in the circulation. Studies have shown that indeed targeting of antigen-specific antibodies are possible by using a monomeric-antigen-Fc fusion [14, 15].

Here, we designed and evaluated a myelin oligodendrocyte glycoprotein-Fc fusion (MOG-Fc) protein to target both MOG-specific T cells and autoantibodies in mouse models of CNS autoimmunity. We fused the extracellular fragment of the MOG protein to the IgG Fc region to prolong their bioavailability. Since monomeric antigens may not efficiently bind low-affinity antibodies, we expressed MOG-Fc fusion protein as a dimer to allow the efficient binding of autoantibodies. Further, we introduced several mutations in the Fc region to ablate complement binding as well as Fc-γ-dependent and antibody-dependent cell-mediated cytotoxicity [16–19]. Using this new reagent, we demonstrate that MOG-Fc fusion proteins selectively eliminate MOG-specific autoantibodies, induce T cell tolerance, and protect mice from MOG-induced EAE.

## Materials and methods

### Fc fusion proteins

We designed an Ig fusion construct (mouse MOG (1–118, N31Q)-linker-IgG2c (C220S, LALA-PG), shortly MOG-Fc) consisting of mouse IgG2c (215–452) together with mouse MOG. We also designed a fusion construct using mouse IgG2c (215–452) and anti-influenza B llama single-domain antibody heavy chain [20] which we used as a control (shortly SD-Fc). We introduced the following mutations in the IgG2c to abolish complement and FcγR receptor binding: L234A, L235A and P329G (LALA-PG), C220S and L351Q. We also mutated the N-glycosylation site (N31Q) in mouse MOG 1–118. The GGGGSGGGGS linker was used to fuse MOG (1–118) and IgG2c-Fc portions. Igk secretion signal was introduced at the N-terminus and His-Tag and AviTag sequences were introduced at the C-terminus to facilitate the purification and multimerization. DNA fragments (Additional file 4) were synthesized by ThermoFisher Scientific as GeneArt gene fragments and cloned into either pTT5 [21] or PB-T vector [22] using NEBuilder^®^ HiFi DNA Assembly (New England Biolabs). The proteins were expressed and purified the IgG-Fc proteins in HEK cells using either a transient transfection [21] or from a transposase-based stable expression system [22] by the core facility of the Max Planck Institute of Biochemistry. The dimeric nature of the purified proteins was confirmed by reducing SDS-PAGE and by mass spectrometry by the core facility of the Max Planck Institute of Biochemistry. Monomeric MOG protein (without Fc) was produced either in HEK cells or in E. coli as recombinant proteins.

### SDS-PAGE

MOG-Fc, SD-Fc, and MOG-specific IgG1 (clone 8.18C5) proteins were denatured in SDS-containing loading buffers (95 °C, 5 min) and loaded (2 μg per lane) onto a gradient (7–15%) polyacrylamide gel under reducing (2 X Laemmli sample buffer; Sigma) or non-reducing (4X Laemmli sample buffer; Bio-Rad) conditions. Two protein markers were used as a protein size reference: Precision Plus Protein standards (Bio-Rad) and PageRuler Plus Prestained Protein Ladder (Thermo Fisher). Gel imaging was performed using a ChemiDoc MP Imaging System (Bio-Rad).

### Mice

Wild-type (WT) C57BL/6, WT SJL/J, CD45.1 C57BL/6, CD45.2 SJL/J, IgH^MOG^ SJL/J, IgH^MOG^ C57BL/6 [23], TCR^1640^ SJL/J (RR) [24] and OSE [23] mice were bred and housed at the animal facilities of the Max Planck Institute of Biochemistry. Mating pairs were fed regular chow ad libitum. Mice were given autoclaved drinking water ad libitum. All animal procedures were performed following the guidelines of the Committee on Animals of the Max Planck Institute of Biochemistry and with approval from the Regierung von Oberbayern (Munich, Germany).

### Serum collection

Blood was collected by retro-orbital bleeding into serum gel tubes (Sarstedt), allowed to stand at room temperature (RT) for 1 h, then centrifuged (10,000 rpm, 5 min, 4 °C) to collect serum. For all kinetics experiments, blood was collected at the time points indicated in the respective graphs. For antibody depletion experiments, asymptomatic RR mice (8–16 weeks of age) were pre-selected based on high anti-MOG IgG titers in their sera. On day 0, 200 µg of MOG-Fc or SD-Fc were administered i.p, following which blood was collected at the indicated time points. Sera were frozen at − 20 °C until their use in ELISA.

### Cell isolation and flow cytometry

Single-cell suspensions were prepared from the spleen and the lymph nodes by mechanical disruption using 40-µm cell strainers (Corning or Fisher Scientific). Cells were collected in RPMI (RPMI 1640, Sigma) containing 10% heat-inactivated Fetal Bovine Serum (FBS, Sigma). Spleen cells were further resuspended in erythrocyte lysis buffer (0.83% NH_4_Cl) and incubated for 3 min at room temperature. The lysis buffer was then neutralized with RPMI containing 10% FBS and the cells were washed and collected in flow cytometry buffer for staining.

Leukocytes from the brain and spinal cord were isolated using a Percoll (GE Healthcare) gradient centrifugation. In brief, mice were perfused with PBS, the CNS tissues were collected and forced through 100-µm cell strainers, and washed in RPMI-1640 containing 10% FBS. The pellet was resuspended in 5 ml of serum-free RPMI-1640, mixed with 2.16 ml Percoll (density 1.123 g/ml), and overlaid onto 5 ml of Percoll (density 1.08 g/ml). After centrifugation at 1200 *g* for 30 min, the cells at the interface were collected and washed with RPMI-1640 containing 10% FBS.

In samples where cytokine expression was not analyzed, cells were directly stained after isolation. In samples that were analyzed for cytokine expression, cells were activated with 50 ng/ml PMA and 500 ng/ml ionomycin in the presence of 5 µg/ml brefeldin A for 4 h at 37 °C before staining. For detection of cell surface markers, cells were washed twice with flow cytometry buffer (PBS containing 1% bovine serum albumin (BSA; Carl-Roth) and 0.1% NaN_3_) and stained with the following antibodies: anti-CD4 (RM4-5), anti-CD45 (30-F11), anti-CD45.1 (A20), anti-CD45.2 (104), anti-CD19 (1D3), anti-CD11c (N418), anti-CD5 (53–7.3), anti-CD21 (7G6), anti-PD-L1 (MIH5), anti-PD-L2 (122), anti-VISTA (MH5A), anti-CD86 (GL1), anti-CD83 (Michel-19), anti-IA/IE (M5/114.15.2), anti-LAG3 (C9B7W), anti-Vα3.2 (RR3-16), anti-Vβ11 (RR3-15), anti-IgMa (DS-1), anti-PD1 (J43) and anti-CD25 (PC61). Fixable viability dye eFluor-780 (Thermo Fisher Scientific) was used at a 1:1000 concentration. Cells were then washed twice in flow cytometry buffer and either resuspended in flow cytometry buffer for acquisition or used for intracellular staining.

For intracellular/intranuclear staining, surface-stained cells were fixed and permeabilized by incubation with 100 µl of Fixation/Permeabilization Buffer (Transcription factor staining set, eBioscience). Cells were then stained with the following antibodies: anti-IFNγ (XMG1.2), anti-IL-17A (TC11-18H10.1), and anti-FoxP3 (FJK-16s). Finally, cells were washed twice in Permeabilization Buffer and resuspended in flow cytometry buffer for acquisition.

Antibodies were purchased from BD Biosciences, Biolegend, or Pharmingen. Antibodies were used in conjugation with one of the following fluorophores: FITC, PE, PerCP-Cy5.5, PeCy7, APC, APC-Cy7, BV421, eFluor450, eFluor780, BV605, BV711, BV785, or APC-R700. Stained samples were acquired on FACS Canto (BD Biosciences). Analysis was performed using FlowJo (TreeStar) software.

### In vivo B cell frequency analysis

Leukocytes from the spleen and mesenteric lymph nodes of IgH^MOG^ SJL/J mice were transferred intravenously (i.v.) at a concentration of 10 million cells/mouse, into CD45.2 SJL/J mice. 200 µg of MOG-Fc or SD-Fc was administered intraperitoneally (i.p.) one day later. The percentage of transferred CD45.1^+^ CD19^+^ B cells was measured by flow cytometry from the spleen and lymph nodes 3 days later.

### Immune cell functionality analysis

To characterize MOG-specific B cell functionality, IgH^MOG^ C57BL/6 mice were injected i.p. with 200 µg of MOG-Fc or SD-Fc. Lymph node cells were isolated 3 days later for flow cytometry.

To characterize CD4 T cell functionality and non-specific APC functionality, splenocytes from OSE mice were transferred i.v. at a concentration of 3–4 million cells/mouse, into WT C57BL/6 mice, along with an i.p. injection of 200 µg of MOG-Fc or SD-Fc. Lymph node cells were isolated 3 days later for flow cytometry.

### EAE

WT C57BL/6 were injected subcutaneously with 200 μl of emulsion containing 100 μg of MOG 35–55 peptide and 500 μg *M. tuberculosis* strain H37 Ra (Difco) in incomplete Freund’s adjuvant (Difco). Mice additionally received 400 ng pertussis toxin (Sigma) i.p. on days 0 and 2 after immunization. 100 µg MOG-Fc or SD-Fc were administered i.p. on days 10, 12, 14, and 16 post EAE induction. Clinical signs of EAE were assessed according to the standard scoring scheme [23]: score 0, healthy; 1, flaccid tail; 1.5, flaccid tail and impaired righting reflex; 2, impaired righting reflex and hind limb weakness; 2.5, one hind leg paralyzed; 3, both hind legs paralyzed with residual mobility in both legs; 3.5, both hind legs completely paralyzed; 4, both hind legs completely paralyzed and beginning front limb paralysis; and 5, moribund or death of the animal after preceding clinical disease. In some cases, the animals were killed before reaching the maximal disease scores in accordance with the animal license regulations.

### In vitro proliferation assay

Single-cell suspensions of splenocytes from OSE mice were washed once with PBS and then subjected to B cell isolation (B cell isolation kit, Biolegend). Purified B cells were loaded with MOG-Fc or SD-Fc (10 µg protein per million cells) for 1 h at 37 °C, washed in RPMI containing 10% FBS, and plated in 96-well U-bottom plates (5 × 10^5^ cells/well) and left overnight at 37 °C. The next day, CD4^+^ T cells were isolated from OSE mice using a CD4 T cell isolation kit (Biolegend). The isolated cells were labeled with 5 µM cell proliferation dye eFluor450 in PBS for 10 min at RT, washed in RPMI containing 10% FBS, and added to the cultured B cells at the concentration of 5 × 10^5^ cells per well. The plates were incubated at 37 °C for 60 h, then stained and analyzed by flow cytometry.

### In vivo proliferation assay

*Protocol 1* Single-cell suspensions of splenocytes from OSE mice were washed once with PBS and then subjected to B cell isolation (B cell isolation kit, Biolegend). The B cells were loaded with MOG-Fc or SD-Fc (10 µg protein per million cells) for 1 h at 37 °C, after which they were washed in PBS and injected i.v. into WT C57BL/6 mice (5–10 million cells per mouse). After 24 h, single-cell suspensions of splenocytes from OSE mice were washed once with PBS and then subjected to CD4^+^ T cell isolation (CD4 T cell isolation kit, Biolegend). The isolated cells were labeled with 5 µM Cell Proliferation Dye eFluor450 in PBS for 10 min at RT, washed in PBS, and injected i.v. into WT C57BL/6 mice (6–8 million cells per mouse). The spleen and lymph node cells were stained and analyzed by flow cytometry on day 6 after T cell transfer.

*Protocol 2* Single-cell splenocyte suspension from OSE mice was washed in PBS and stained in 5 µM cell proliferation dye eFluor450 in PBS for 10 min at RT. The cells were washed in PBS and injected i.v. into CD45.1 C57BL/6 mice (20–30 million cells per mouse). 2 days after cell transfer, mice were injected i.p. with 200 μg of MOG-Fc or SD-Fc in 200 μl PBS. Six days after cell transfer, the spleen, and lymph node cells were stained and analyzed by flow cytometry.

### Enzyme-linked immunosorbent assay (ELISA)

ELISAs were performed with the following general considerations. 96-well Maxisorp Nunc-immuno plates (Thermo Scientific) were coated overnight at 4 °C, PBS-T (0.1% Tween 20 in PBS) was used for washing the plates, and blocking was done with 10% FCS in PBS for one hour in room temperature. Incubations with serum samples, antibodies, and streptavidin were all performed in the blocking buffer at room temperature. TMB (3,3ʹ,5,5ʹ-tetramethylbenzidine) or ABTS (2,2ʹ-azinobis [3-ethylbenzothiazoline-6-sulfonic acid]-diammonium salt) substrate solution (activated with 0.1% H_2_O_2_) (Biolegend) was used for detection. TMB reaction was stopped with 1N H_2_SO_4_. Plates were measured in a spectrophotometer (Perkin Elmer) at 450 nm (for TMB) or 405 nm (for ABTS) after 10 min.

#### MOG-specific antibody detection

Plates were coated with 20 µg/ml of MOG, MOG-Fc, or SD-Fc in PBS (100 µl per well), washed, blocked for one hour, washed again, and incubated with serum samples at different dilutions for 2 h at RT. The MOG-specific antibody 8.18C5 and mouse IgG1 (Biolegend) were used in a dilution series as standards. After washing, biotin-labeled anti-mouse IgG (5 ng/ml; SouthernBiotech) was added for 1 h, then washed and incubated with HRP-labeled streptavidin (0.5 µg/ml; Biolegend) for another hour. After final washing, the signal was developed by adding a substrate solution and measured in a spectrophotometer.

#### Residual MOG-Fc ELISA

Plates were coated with 1 µg/ml of anti-His-tag antibody (Biolegend, 100 µl per well), washed, blocked for one hour, washed again, and incubated with serum samples for 2 h at RT. MOG-Fc and MOG were used as standards. After washing, a biotin-labeled 8.18C5 antibody (1 µg/ml; produced in-house) was added for 1 h, then washed and incubated with HRP-labeled streptavidin (0.5 µg/ml; Biolegend) for another hour. After final washing, the signal was developed by adding a substrate solution and measured in a spectrophotometer.

### MOG binding assay

AviTag-containing MOG-Fc and monomeric MOG proteins were biotinylated using BirA biotin-protein ligase kit (BirA-500, Avidity LLC) according to the manufacturer’s instructions and conjugated with APC-labeled streptavidin to form tetramers. Splenocytes from wild-type and IgH^MOG^ C57BL/6 mice were mixed in different ratios (100, 50, 25, 12.5, 6.2, and 0 percent of IgH^MOG^ cells) and stained with equimolar amounts of MOG-Fc or MOG tetramers, as well as with anti-CD19 (6D5, Biolegend) and Fixable viability dye eFluor 780 (Thermo Fisher Scientific). Stained samples were acquired on FACS Canto (BD Biosciences). Analysis was performed using FlowJo (TreeStar) software.

### Statistical analysis

GraphPad Prism 9 (GraphPad Software, Inc.) was used for all statistical analyses. Information on statistical tests used for analysis is mentioned in figure legends. *P* values below 0.05 were considered significant. Bars depict the mean ± standard error of the mean.

## Results

### Design, production, and characterization of MOG-Fc fusion protein

**Fig. 1:**
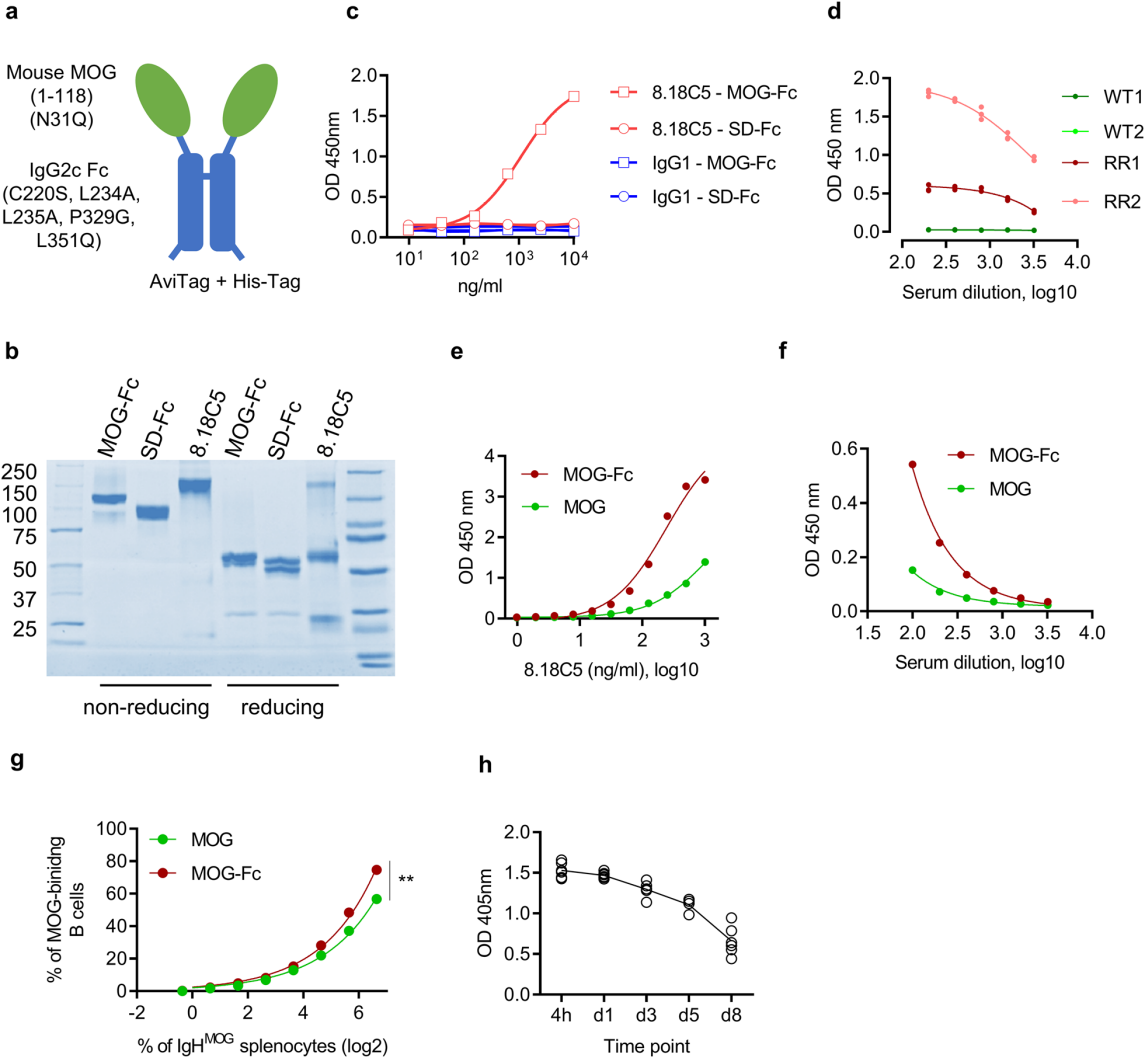
Design, production, and characterization of MOG-Fc fusion protein. **a** Schematic representation of MOG-Fc fusion protein. The extracellular domain of the mouse MOG protein (amino acids 1–118) is depicted in green and the Fc portion of the mouse IgG2c is depicted in blue. The mutations to abolish glycosylation of MOG protein and to disrupt complement and Fc receptor binding sites are indicated within brackets. His-Tag and AviTag sequences were introduced at the C-terminus for purification and detection. **b** SDS-PAGE (both reducing and non-reducing) of the purified MOG-Fc, SD-Fc proteins, and MOG-specific antibody 8.18C5. **c** Dose-dependent binding analysis of 8.18C5 and control IgG1. ELISA plates were coated with MOG-Fc or SD-Fc and serially diluted 8.18C5 or IgG1 were added. OD at 450 nm is shown. **d** Binding of MOG-Fc to serum anti-MOG antibodies. Serum from 2 individual WT SJL/J and RR mice were used (*n* = 3) for the detection of MOG antibodies by ELISA. OD at 450 nm is shown. **e** Comparison of the binding efficiencies of MOG-Fc and monomeric MOG to 8.18C5. MOG-Fc or monomeric MOG was coated and detected using serially diluted anti-MOG monoclonal antibody 8.18C5. OD at 450 nm is shown. **f** Comparison of the binding of MOG-Fc and monomeric MOG to serum anti-MOG antibodies from RR mice. OD at 450 nm is shown. **g** Detection of MOG-specific B cells by MOG-Fc and monomeric MOG. MOG-Fc and monomeric MOG were biotinylated at their AviTag and tetramerized with a streptavidin-coupled fluorochrome. IgH^MOG^ splenocytes were mixed with wild-type mouse splenocytes at various ratios and used for the staining with tetramerized MOG-Fc or monomeric MOG proteins. Samples were analyzed by flow cytometry and the percentage of MOG binding B cells was shown. **P = 0.0078 (Wilcoxon matched-pairs signed rank test). **h** Residual MOG-Fc or SD-Fc in WT SJL/J mice after a single injection. 200 µg of MOG-Fc or SD-Fc was injected into the mice (*n* = 6 per group) and sera were collected after 4 h and on days 1, 3, 5, and 8 post-injections. Residual-MOG ELISA was performed and the OD at 405 nm is shown. All experiments were performed at least twice

To demonstrate the feasibility of simultaneous targeting of antigen-specific T cells and B cells, we engineered a myelin antigen MOG-Fc fusion protein. We fused the extracellular domain (amino acids 1–118) of mouse MOG protein to an Fc region consisting of mouse IgG2c (amino acids 215–452; EU numbering) to prolong the systemic bioavailability of the fusion protein (Fig. 1a). We also generated an Fc fusion construct using a similar-sized protein (anti-influenza B llama single-domain antibody heavy chain [20]—hereafter referred to as SD-Fc) as a control. We chose mouse IgG2c since this subclass is expressed in mice carrying the IgH-1b haplotype (C57BL/6, C57BL/10, SJL, and NOD) [25]. Since the IgG2a and IgG2c subclasses are seen as functionally comparable, and the most active among the subclasses to bind complement [26], we introduced several mutations in the Fc portion to abolish complement and Fc receptor binding. The mutations that were introduced to the fusion constructs are as follows: C220S: to remove the cysteine that typically pairs with a light chain; L234A, L235A, P329G (LALA-PG): to eliminate complement binding and fixation as well as Fc-γ-dependent and antibody-dependent cell-mediated cytotoxicity [16–18]; L351Q: to abolish complement binding [19]. The N-glycosylation of MOG has been previously shown to affect antibody binding. To avoid glycosylation-dependent effects on the binding and depletion efficiency of the Fc fusion construct, we also introduced a mutation at the single N-glycosylation site in MOG (N31Q) (Fig. 1a).

We expressed and purified these fusion proteins in mammalian cells to preserve their native conformation. We confirmed the expression of the fusion protein by SDS-PAGE as well as mass spectrometry (Fig. 1b, Additional file 1: Figure S1a). To confirm that the MOG-Fc is folded correctly and is recognized by MOG-specific antibodies, we performed an enzyme-linked immunosorbent assay (ELISA) using the MOG-specific (conformation-specific) monoclonal antibody 8.18C5 as well as sera from relapsing–remitting (RR) mice [24], which contain polyclonal MOG-specific antibodies that arose spontaneously and are essential for autoimmune disease development. While MOG-specific 8.18C5 monoclonal antibody recognized the MOG-Fc but not SD-Fc protein in a dose-dependent manner, a control IgG antibody neither detected MOG-Fc nor SD-Fc protein (Fig. 1c, Additional file 1: Figure S1b). This suggested that the MOG-Fc fusion protein is folded correctly and is only specifically detectable by MOG-specific antibodies. Similarly, serum from RR mice but not wild-type (WT) mice recognized MOG-Fc protein (Fig. 1d). A comparative analysis of the binding efficiency of the dimeric MOG-Fc construct with the monomeric MOG without Fc fusion protein by 8.18C5 and RR mouse serum suggested that MOG-Fc was considerably better in binding to MOG-specific antibodies than monomeric MOG protein (Fig. 1e, f). Furthermore, staining of IgH^MOG^ B cells which express a heavy chain of the MOG-antibody 8.18C5 [23] showed that the MOG-specific B cells bind to MOG-Fc better than to monomeric MOG (Fig. 1g). Next, we characterized the in vivo bioavailability of MOG-Fc. We injected WT SJL/J mice with a single dose of MOG-Fc and measured the residual protein levels in the serum by ELISA. We observed that MOG-Fc persists in the serum for at least 8 days post-injection (Fig. 1h). We also validated this observation in WT C57BL/6 mice, and further found that MOG-Fc also persists longer than monomeric MOG (Additional file 1: Figure S1c). In summary, the dimeric MOG-Fc is specifically bound by anti-MOG antibodies, recognized by MOG-specific B cells, and persists longer in vivo than monomeric MOG.

### MOG-Fc depletes MOG-specific antibodies but spares MOG-reactive B cells

**Fig. 2:**
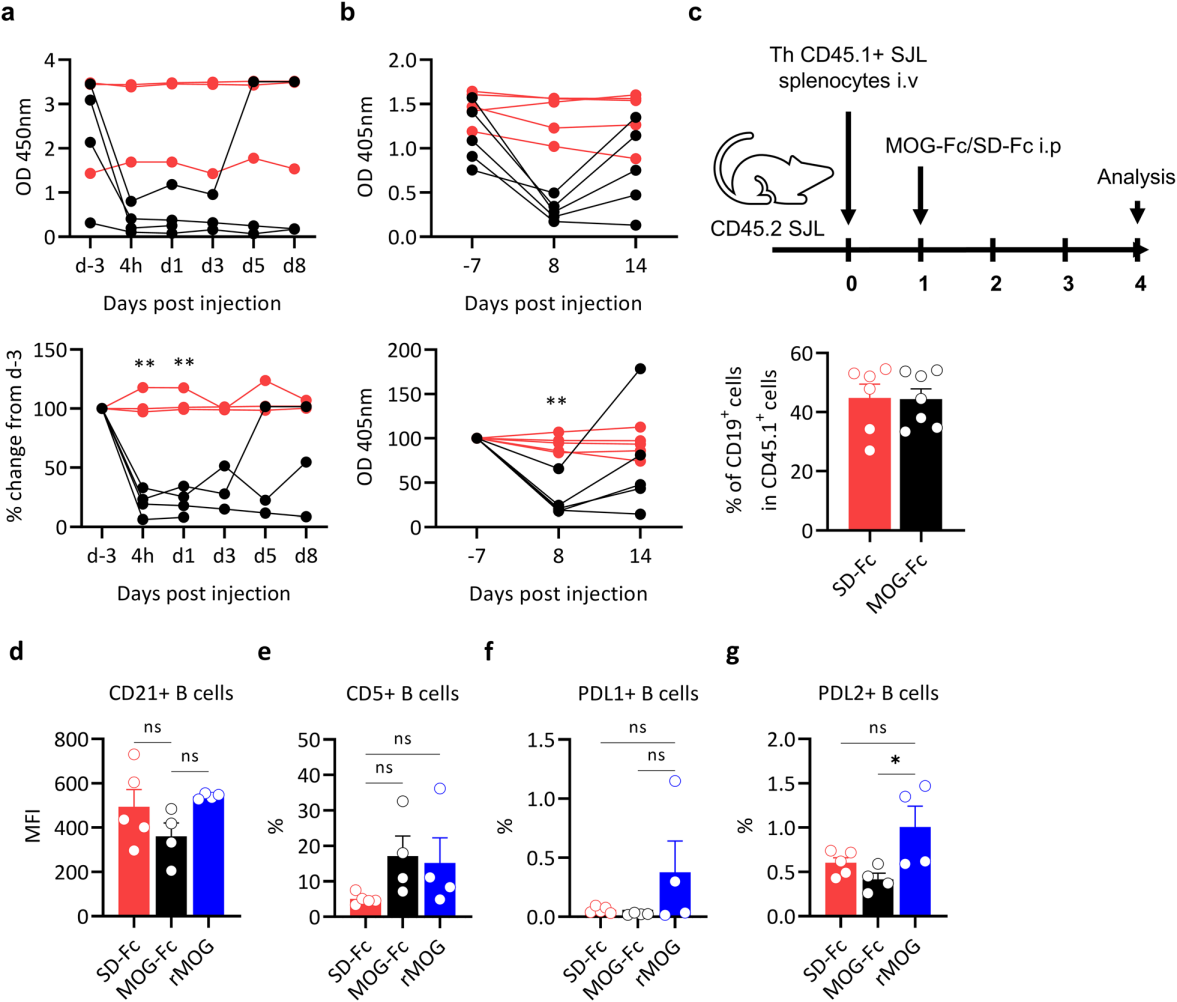
MOG-Fc depletes anti-MOG antibodies but spares MOG-reactive B cells. **a** Kinetics of the depletion of anti-MOG antibodies in the serum of RR mice injected with MOG-Fc (*n* = 4) or SD-Fc (*n* = 3); RR mice were injected with 200 µg of MOG-Fc or SD-Fc and the sera were collected 3 days before injection, 4 h after injection, and on days 1, 3, 5 and 8 after injection. anti-MOG IgG ELISA was performed and OD at 450 nm (top panel), and percent change compared to day -3 (bottom panel) are shown. Each circle represents one mouse. **P = 0.0021 at 4 h, **P = 0.0012 at day 1 (Sidak’s multiple comparison test). **b** Depletion of anti-MOG antibodies in the serum of RR mice injected with MOG-Fc (*n* = 5), SD-Fc (*n* = 5). RR mice were injected with 200 µg of MOG-Fc or SD-Fc and the sera were collected 7 days before injection and on days 8 and 14 after injection. anti-MOG IgG ELISA was performed and OD 405 nm (top panel), and percent change compared to day -7 (bottom panel) are shown. One representative experiment out of 3 experiments performed is shown. Each circle represents one mouse. **P = 0.0026 (Sidak’s multiple comparison test). **c** Splenocytes from CD45.1^+^ IgH^MOG^ SJL/J mice were transferred i.v. to CD45.2^+^ SJL/J mice which were subsequently injected with 200 µg of MOG-Fc (*n* = 6) or SD-Fc (*n* = 6). Experimental setup (top panel) and the frequencies of B cells (bottom panel) among the transferred CD45.1^+^ cells analyzed by flow cytometry 3 days after MOG-Fc and SD-Fc injection were shown. Each circle represents one mouse. Data from 2 experiments are pooled. Data are represented as mean ± s.e.m. **d–g** IgH^MOG^ C57BL/6 mice received 200 µg of MOG-Fc (*n* = 4) or SD-Fc (*n* = 5) or rMOG (*n* = 4). After 3 days, lymph node cells were analyzed by flow cytometry. Each circle represents one mouse. The Mean Fluorescence Intensity (MFI) of CD21 (**d**), frequencies of CD5^+^ cells (**e**), frequencies of PDL1^+^ cells (**f**), and frequencies of PDL2^+^ cells (**g**) in IgH^MOG^ B cells are shown. All data are represented as mean ± s.e.m. All experiments were performed at least twice

Having confirmed the specificity of MOG-Fc in binding to MOG-specific antibodies in vitro, we sought to determine their ability to neutralize and clear MOG-specific autoantibodies in vivo. We injected RR mice (which have high titers of circulating MOG-specific antibodies) with a single dose of MOG-Fc or SD-Fc and measured their serum autoantibody levels by ELISA. We noted that a single injection MOG-Fc was sufficient to significantly reduce the circulating antibodies within 4 h while no reduction of antibodies in SD-Fc treated mice (Fig. 2a). Further, the reduced MOG-specific antibody levels in the serum persisted for at least 8 days after injection in most of the mice, correlating with our observation that MOG-Fc remains in circulation for at least 8 days post-injection (Fig. 2a, b). Subsequently, the MOG-specific antibodies reappeared and reached pre-injection levels around day 14 after injection (Fig. 2b).

While the introduction of mutations silenced some effector functions of the Fc fusion protein, MOG-Fc still efficiently binds to MOG-specific B cells (Fig. 1g). Hence, we examined whether MOG-Fc binding would also affect B cell functions. To this end, we transferred CD45.1^+^ MOG-specific B cells into CD45.2^+^ SJL/J mice and then injected these mice with MOG-Fc or SD-Fc (Fig. 2c). The analysis of the lymph nodes after 4 days showed comparable frequencies of transferred MOG-specific B cells between MOG-Fc and SD-Fc injected mice, indicating that MOG-Fc did not deplete MOG-specific B cells (Fig. 2c).

Since antigen stimulation in the absence of inflammatory stimuli may lead to tolerance, which is reflected by the expression patterns of co-stimulatory and co-inhibitory molecules, we analyzed the expression of some co-stimulatory/co-inhibitory markers that are known to be associated with B cell functionality in autoimmunity. We administered a single dose of MOG-Fc or SD-Fc to IgH^MOG^ mice and analyzed the lymph node B cell compartment after 72 h. We found no significant differences in the expression of regulatory B cell markers CD5 and CD21 [27]. We observed that while there was marginal reduction in the frequencies of PD-L1 and PD-L2^+^ cells (both of which bind PD1 on T cells to negatively regulate T cell effector functions [28]), the differences in PDL1 and PDL2 expression on B cells were not significant. We also found no significant differences in the expression of B cell regulatory markers on administration of monomeric MOG, except for a slight increase in PD-L2 + B cell frequencies (Fig. 2d–g). Also, there were no detectable changes in the expression levels of MHC class II and VISTA expression in B cells between MOG-Fc and SD-Fc-treated mice (Additional file 2: Fig. S2a, b).

Having observed no significant changes in MOG-specific B cell functionality, we next investigated whether MOG-Fc could affect other non-antigen-specific antigen-presenting cells (APCs). We thus administered a single dose of MOG-Fc or SD-Fc to WT C57BL/6 mice and analyzed both the B cells and CD11c^+^ cells in the lymph nodes by flow cytometry. The expression of the co-stimulatory markers CD86, CD83, PDL1, PDL2, VISTA, and IA/IE (MHC class II) was not altered by MOG-Fc treatment, indicating that MOG-Fc does not affect non-antigen-specific APCs (Additional file 2: Figure S2a, b). Taken together, these results indicated that MOG-Fc treatment does not have a significant impact on antigen-specific or non-specific APC functionality.

### MOG-Fc does not affect APC functionality

**Fig. 3:**
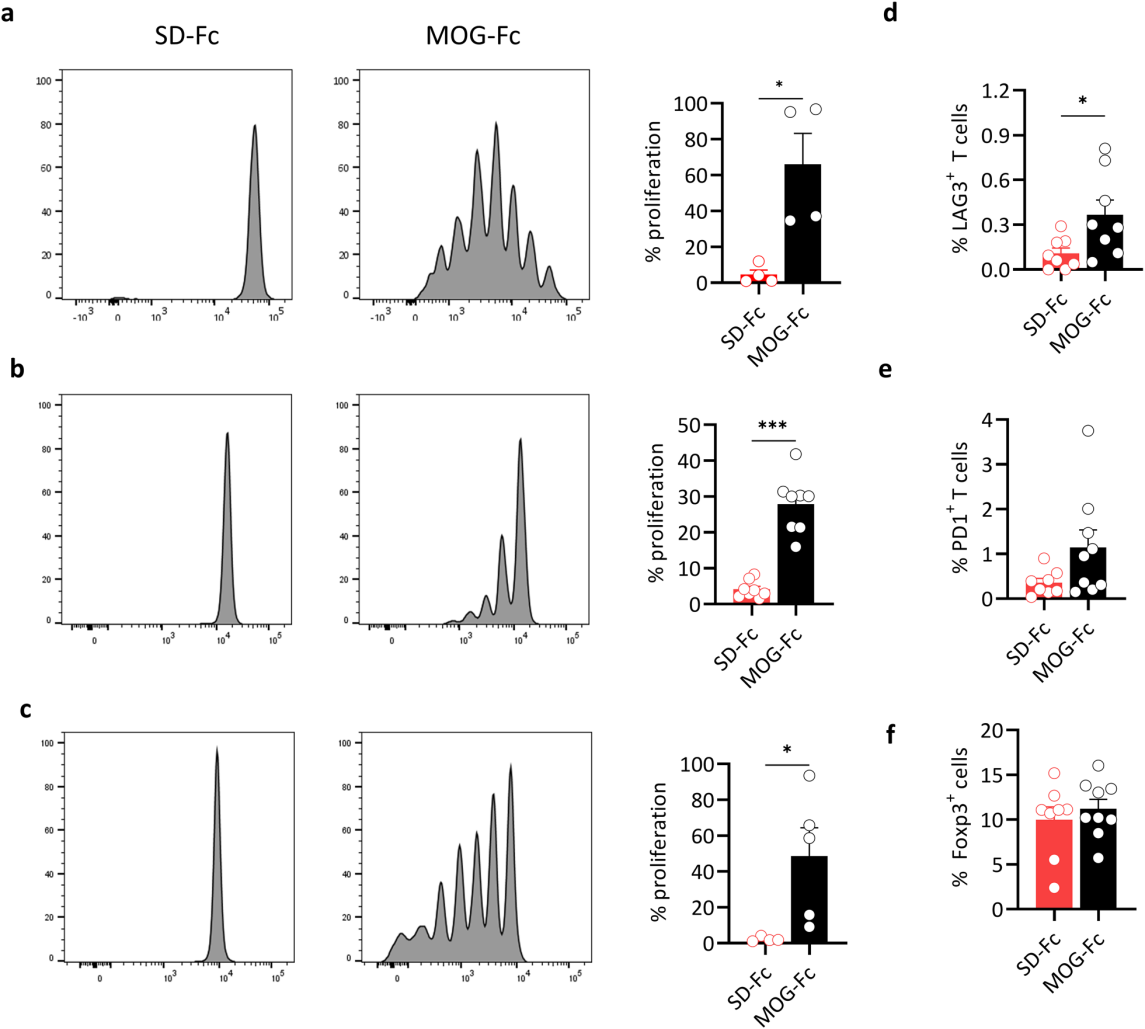
Effect of MOG-Fc on antigen presentation and T cell functionality. **a** Proliferation of MOG-specific CD4^+^ T cells that were cocultured with IgH^MOG^ B cells preloaded with MOG-Fc or SD-Fc (*n* = 4 per group). Representative flow cytometry plots (left panel) and the percentage of proliferating T cells (right panel) are shown. *P = 0.0286 (Mann–Whitney’s U test). **b** The proliferation of MOG-specific CD4^+^ T cells in vivo in the presence of antigen preloaded IgH^MOG^ B cells. WT C57BL/6 mice received (i.v.) IgH^MOG^ B cells preloaded with MOG-Fc (*n* = 8) or SD-Fc (*n* = 8), and one day later they received (i.v.) CD4^+^ T cells from OSE mice. Representative flow cytometry plots (left panel) and the percentage of proliferating T cells (right panel) are shown. Each circle represents one mouse. Data from 2 experiments are pooled. ***P = 0.0002 (Mann–Whitney’s U test). **c** The proliferation of MOG-specific CD4^+^ T cells in vivo in the presence of soluble MOG antigen and IgH^MOG^ B cells. WT C57BL/6 mice received (i.v.) OSE splenocytes cells and 2 days later they were injected (i.p.) with 200 µg of MOG-Fc (*n* = 5) or SD-Fc (*n* = 5). Representative flow cytometry plots (left panel) and the percentage of proliferating T cells (right panel) are shown. Each circle represents one mouse. Data from 2 experiments are pooled. ***P = 0.0159 (Mann–Whitney’s U test). **d–f** WT C57BL/6 mice received (i.v.) OSE splenocytes along with 200 µg of MOG-Fc (*n* = 9) or SD-Fc (*n* = 8). After 3 days, lymph node cells were analyzed by flow cytometry. Each circle represents an individual mouse. Data from 2 experiments are pooled. **d** The frequencies of LAG3^+^ cells as a percentage of MOG-specific CD4^+^ T cells. *P = 0.0351 (unpaired T-test). **e** The frequencies of PD1^+^ cells as a percentage of MOG-specific CD4^+^ T cells; **f** the frequencies of Foxp3^+^ cells as a percentage of total CD4^+^ T cells. All data are represented as mean ± s.e.m. All experiments were performed at least twice

We have previously shown that MOG-specific B cells are potent antigen-presenting cells that are crucial to drive T cell proliferation and induce spontaneous autoimmune disease [23]. To identify if MOG-Fc affected the antigen-presenting ability of B cells, we performed an in vitro proliferation assay. We first loaded MOG-specific B cells with MOG-Fc or SD-Fc, after which we cultured them with a fluorochrome (eFlour450) labeled MOG-specific (2D2) CD4^+^ T cells [23]. Flow cytometry analysis showed that the T cells proliferated significantly under MOG-Fc than SD-Fc, indicating that the MOG-specific B cells were able to capture, process, and present MOG-Fc to the T cells (Fig. 3a). We subsequently investigated whether MOG-specific B cells can present MOG-Fc in vivo. To this end, we transferred MOG-specific B cells loaded with MOG-Fc or SD-Fc into WT C57BL/6 mice, and 24 h later, these mice were also given eFlour450-labeled 2D2 T cells. We measured 2D2 T cell proliferation after 6 days by flow cytometry and found that 2D2 T cells from mice that received MOG-Fc but not SD-Fc loaded B cells showed significant proliferation, indicating that the B cells are capable of presenting MOG-Fc to T cells in vivo (Fig. 3b). Additionally, we wanted to verify if the MOG-Fc in circulation can also be taken up and presented by the MOG-specific B cells. To this end, we transferred eFlour450-labeled splenocytes from opticospinal encephalomyelitis (OSE) mice which have both MOG-specific T and B cells into CD45.1 C57BL/6 mice and then injected them MOG-Fc or SD-Fc. In this system too, the transferred T cells proliferated significantly under the MOG-Fc stimulus, suggesting that the B cells can take up circulating MOG-Fc and present it to the T cells (Fig. 3c). These observations indicated that the antigen-presentation capacity of B cells was unaffected, and in addition, the ability of T cells to get activated and proliferate was also unaffected by MOG-Fc.

### MOG-Fc treatment affects MOG-specific T cell functionality

We sought to study the effect of MOG-Fc treatment on MOG-specific T cells as CD4^+^ T cells are the primary drivers of CNS autoimmunity. Although MOG-Fc does not affect T cell proliferative capacity (Fig. 3a–c), the MOG-Fc could induce additional responses that determine their eventual functionality. We measured the expression of Foxp3, LAG3, and PD1 by MOG-specific T cells upon treatment with MOG-Fc. A single injection of MOG-Fc or SD-Fc into WT C57BL/6 mice which additionally received MOG-specific 2D2 T cells increased the frequencies of LAG3^+^ CD4^+^ T cells among the MOG-specific T cell population (Fig. 3d). There was also a trend toward higher expression of PD1, although it did not reach statistical significance (Fig. 3e). However, we found no difference in the frequencies of Foxp3^+^ T cells (Fig. 3e, f). This suggested that MOG-Fc could induce a potentially tolerogenic T cell response by inducing the LAG3 and PD-1 inhibitory receptors.

### MOG-Fc treatment ameliorates EAE

**Fig. 4:**
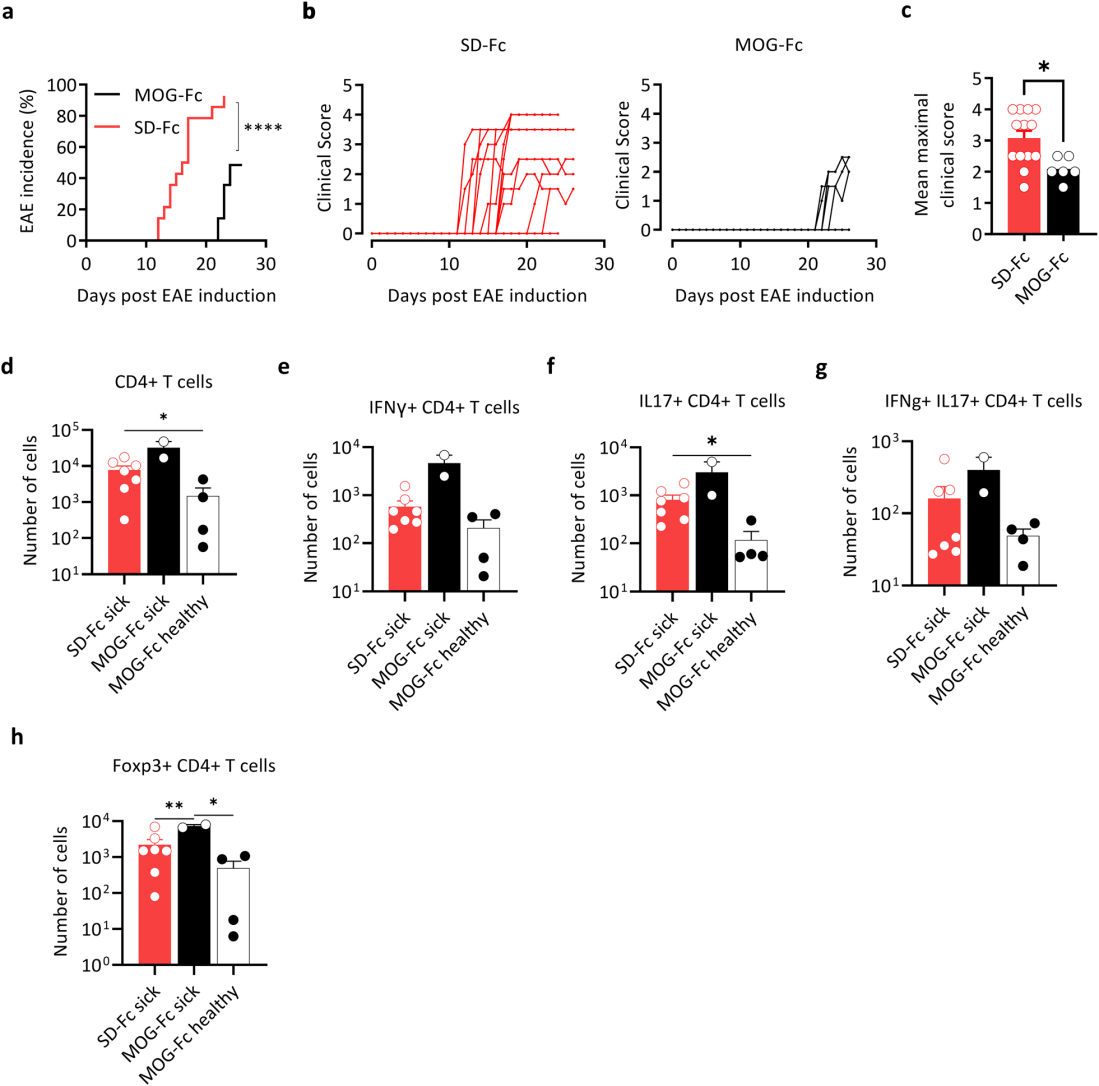
MOG-Fc mitigates EAE in C57BL/6 mice.** a–c** EAE incidence **(a)**, mean clinical score (**b),** mean maximal scores **(c)** after immunization of C57BL/6 mice with MOG 35–55 in CFA (MOG-Fc *n* = 14, SD-Fc *n* = 14). Data from 2 experiments are pooled. ****P < 0.0001 (log-rank Mantel–Cox test). **d–h** The numbers of total (**d**), IFNγ^+^ (**e**), IL-17^+^ (**f**), IFNγ^+^ IL-17^+^ (**g**) and Foxp3^+^ (**h**) CD4^+^ T cells in the spinal cords of healthy (asymptomatic) and sick mice treated with MOG-Fc or SD-Fc (MOG-Fc healthy *n* = 4, MOG-Fc sick *n* = 2, SD-Fc sick *n* = 7). Each circle represents an individual mouse. **d** *P = 0.0359, SD-Fc sick vs MOG-Fc healthy; **f** *P = 0.0164, SD-Fc sick vs MOG-Fc healthy; **h** **P = 0.0082, SD-Fc sick vs MOG-Fc sick, *P = 0.0401, MOG-Fc sick vs MOG-Fc healthy. All data are represented as mean ± s.e.m. An unpaired T-test with Welch’s correction was used for group comparisons

Next, we sought to evaluate the effect of MOG-Fc treatment on the development of autoimmune disease using experimental autoimmune encephalomyelitis (EAE). To this end, we immunized WT C57BL/6 mice with MOG 35–55 peptide and then administered MOG-Fc or SD-Fc starting from day 10 after immunization every 2 days until day 16 (4 doses in total) and monitored these mice for neurological symptoms. Interestingly, we observed significant protection from EAE development and reduced disease severity in mice treated with MOG-Fc compared to SD-Fc (Fig. 4a–c). None of the MOG-Fc-treated mice showed disease symptoms until day 22 while more than 80% of the SD-Fc-treated mice showed EAE symptoms (Fig. 4a). The maximal disease severity of the MOG-Fc treated mice was also significantly lower than that of SD-Fc treated mice (Fig. 4b–c). Flow cytometry analysis of the CNS infiltrates also showed a similar number of infiltrating CD4^+^ T cells in sick mice, whereas the numbers of CD4^+^ T cells were significantly lower in MOG-Fc-treated mice which remained healthy (Fig. 4d). The total numbers of Foxp3^+^ T cells in the spinal cord were significantly increased in the EAE-affected MOG-Fc-treated mice, and the numbers of IFNγ^+^, IL-17^+^ and IFNγ^+^IL-17^+^ T cells were slightly higher in EAE-affected mice, whereas their numbers were significantly lower in healthy MOG-Fc-treated mice (Fig. 4e–h, Additional file 3: Fig. S3a-c). Hence, we conclude that MOG-Fc efficiently mitigates EAE development and progression in MOG-immunized mice.

## Discussion

Several ways to induce antigen-specific tolerance have been reported in pre-clinical models of autoimmune diseases [5]. These approaches include transplantation of hematopoietic stem cells or modified immune cells expressing autoantigen [7, 29], injection of soluble peptide autoantigen or coated nanoparticles [9, 11, 30], gene vaccination [12, 13, 31]. While these studies have successfully shown that antigen-specific T cell tolerance can be induced via these treatments, there is little evidence that antigen-specific B cells or autoantibodies can also be targeted with these approaches.

Antigen-specific tolerization approaches have also been tested in CNS autoimmune disease models as well as in MS patients, where the effective disease management without globally affecting immune responses remains poorly addressed [32]. Especially, there is no treatment that targets antigen-specific T cells and B cells is currently available. One reason for this is the lack of knowledge of a specific autoantigen that triggers MS. Also, historically MS was viewed as a T cell-driven disease but the role of B cells and antibodies in the pathogenesis is increasingly being appreciated [33]. However, antigen-specific approaches are viable at least in some subgroups of patients with MOG-antibody associated autoimmune CNS demyelination [34]

In this study, we have developed an approach to target both antigen-specific T cells and autoantibodies using a myelin antigen, MOG. We selected the Fc-fusion strategy for several reasons. First, Fc-fusion is commonly used to enhance the bioavailability of biologically active proteins which have a short serum half-life because of their rapid renal clearance [35]. Second, due to their dimeric nature, Fc-fusion may enhance the avidity of antibody binding to the target antigen, which is especially advantageous for targeting low-affinity antibodies. Third, numerous therapeutic Fc fusion proteins have been extensively used for the treatment of immune-mediated diseases.

We fused the extracellular domain of MOG, which carry immunodominant epitopes that drive autoimmune demyelinating disease upon immunization [36] with the Fc region. The Fc constructs included a C220S substitution to remove the cysteine that typically pairs with a light chain. Further, several mutations have been introduced to abolish N-glycosylation, complement binding and fixation. In line with previous reports [14, 37], the serum levels of Fc-fusion protein were much higher than the monomeric MOG protein and detectable up to 8 days after a single injection. This prolonged bioavailability implies MOG-Fc is more suitable than MOG to induce immune tolerance and eliminates the need for vehicles to enhance bioavailability. Besides their extended bioavailability, the MOG-Fc was also significantly better in binding to monoclonal and polyclonal MOG-specific antibodies as well as MOG-specific B cells supporting our notion that dimeric antigen display enhances the avidity of binding by MOG-specific antibodies. The increased bioavailability coupled with enhanced avidity, if translated to a clinical setting, would allow reduced dosage, eliminate the need for adjuvants, and more efficiently deplete MOG-specific antibodies and B cells.

Antigen-specific tolerance is typically induced when soluble antigens are delivered in the absence of inflammatory stimuli. The soluble antigen application has been shown to induce a myriad of tolerogenic immune responses including anergy, induction of regulatory T cells, deletion of antigen-specific immune cells, and skewed cytokine and co-stimulatory molecule expression [5]. Consistent with these observations, we noted that MOG-Fc treatment increased the expression of inhibitory receptors PD-1 and LAG3 expression in MOG-specific T cells which could contribute to the immune tolerance. However, MOG-Fc treatment did not affect the expression of co-stimulatory or co-inhibitory molecules in antigen-presenting cells. Also, the ability of antigen-presenting cells to present antigen was unaffected.

We have evaluated whether the MOG-Fc reagent is suitable for targeting antigen-specific B cells and/or autoantibodies. Unlike the large body of literature that showed antigen-specific T cell tolerization approaches, few studies have shown that antigen-specific B cells or autoantibodies can be targeted for the treatment of autoimmunity. Similar to previous studies [14], MOG-Fc fusion protein specifically depleted anti-MOG antibodies in vivo thus overcoming the limitation of broader immune suppression. Although we observed that MOG-Fc is capable of binding to MOG-specific B cells, it did not deplete these B cells. This additionally supported that the mutations that were introduced in the Fc region indeed eliminated antibody-mediated cytotoxicity. Importantly, prophylactic MOG-Fc treatment substantially protected mice from MOG-induced EAE. MOG-Fc treated mice were less susceptible to the EAE development as long as the treatment could be maintained. The mice that remained healthy after MOG-Fc treatment showed a reduced infiltration of T cells in the CNS consistent with their disease protection. Taken together with our data on the kinetics of antibody depletion by MOG-Fc, multiple doses of MOG-Fc may have to be administered for prolonged disease protection, particularly in models with longer disease duration. In this regard, more experiments with models with longer disease duration—like spontaneous EAE models, are necessary.

Collectively, our data support the contention that autoantigen-Fc fusion protein could be used to blunt antigen-specific T cells and autoantibodies thereby suppressing complex autoimmune responses without compromising systemic autoimmunity. With the possibility to use MOG-Fc for antibody depletion and/or tolerance induction, with further testing this protein can potentially be used in the treatment of MOG-antibody diseases. The development of more such fusion proteins where the target autoantigen is known would also widen the therapeutic possibilities in autoimmune diseases.

### Supplementary Information

**Additional file 1: Figure S1.** Characterization of MOG-Fc and SD-Fc proteins. a. Mass Spectrometric analysis of MOG-Fc and SD-Fc proteins. b. Dose-dependent binding of MOG-Fc to 8.18C5 (*n* = 3—4 replicates). anti-MOG-Fc IgG ELISA was performed and the OD at 450 nm is shown. c. Residual MOG-Fc or monomeric MOG in WT C57BL/6 mice after a single injection. 200 µg of MOG-Fc or monomeric MOG was injected into the mice (*n* = 4 per group) and sera were collected before injection, after 4 h, and on days 1, 3, and 5 post-injections. The serum concentration of MOG-Fc and monomeric MOG proteins is shown.

**Additional file 2: Figure S2. **MOG-Fc does not affect non-antigen-specific APC functionality. a-b. WT C57BL/6 mice received (i.v.) OSE splenocytes along with 200 µg of MOG-Fc (*n* = 9) or SD-Fc (*n* = 8). After 3 days, lymph node cells were analyzed by flow cytometry. Data from 2 experiments are pooled. Each box in the heatmap represents one mouse. a. The frequencies of PDL1^+^, PDL2^+^, PDL1^+^, PDL2^+^, and VISTA^+^ populations in CD11c^+^ cells and B cells are represented as a heatmap. b. The frequencies of CD86^+^, CD83^+^, and IA/IE (MHC II)^+^ populations in CD11c^+^ cells and IA/IE (MHC II)^+^ population in B cells are represented as a heatmap.

**Additional file 3: Figure S3. **Representative flow cytometry plots for CNS infiltrates. a-c. Representative flow cytometry plots from the spinal cord of SD-Fc sick (left), MOG-Fc sick (middle), and MOG-Fc healthy (right) mice. The values on the gates show percentages (of the parent population) a. CD4 staining (gated on CD45^+^ cells). b. Foxp3 staining (gated on CD4^+^ T cells). c. IFNγ and IL-17 staining (gated on CD4^+^ T cells).

**Additional file 4.** Sequences of MOG-Fc and SD-Fc constructs.

## Data Availability

All data relevant to this study are included in the article and its additional information files.

## Abbreviations

CNS: Central nervous system
DNA: Deoxyribonucleic acid
RNA: Ribonucleic acid
EAE: Experimental autoimmune encephalomyelitis
MS: Multiple sclerosis
OSE: Opticospinal encephalomyelitis
RR: Relapsing–remitting
MOG: Myelin oligodendrocyte glycoprotein
