# Supplementary figures and images for: An engineered Fc fusion protein that targets antigen-specific T cells and autoantibodies mitigates autoimmune disease
Source: J Neuroinflammation. 2023 Dec 6;20:291. doi: 10.1186/s12974-023-02974-9 (PMC10702099; doi:10.1186/s12974-023-02974-9)

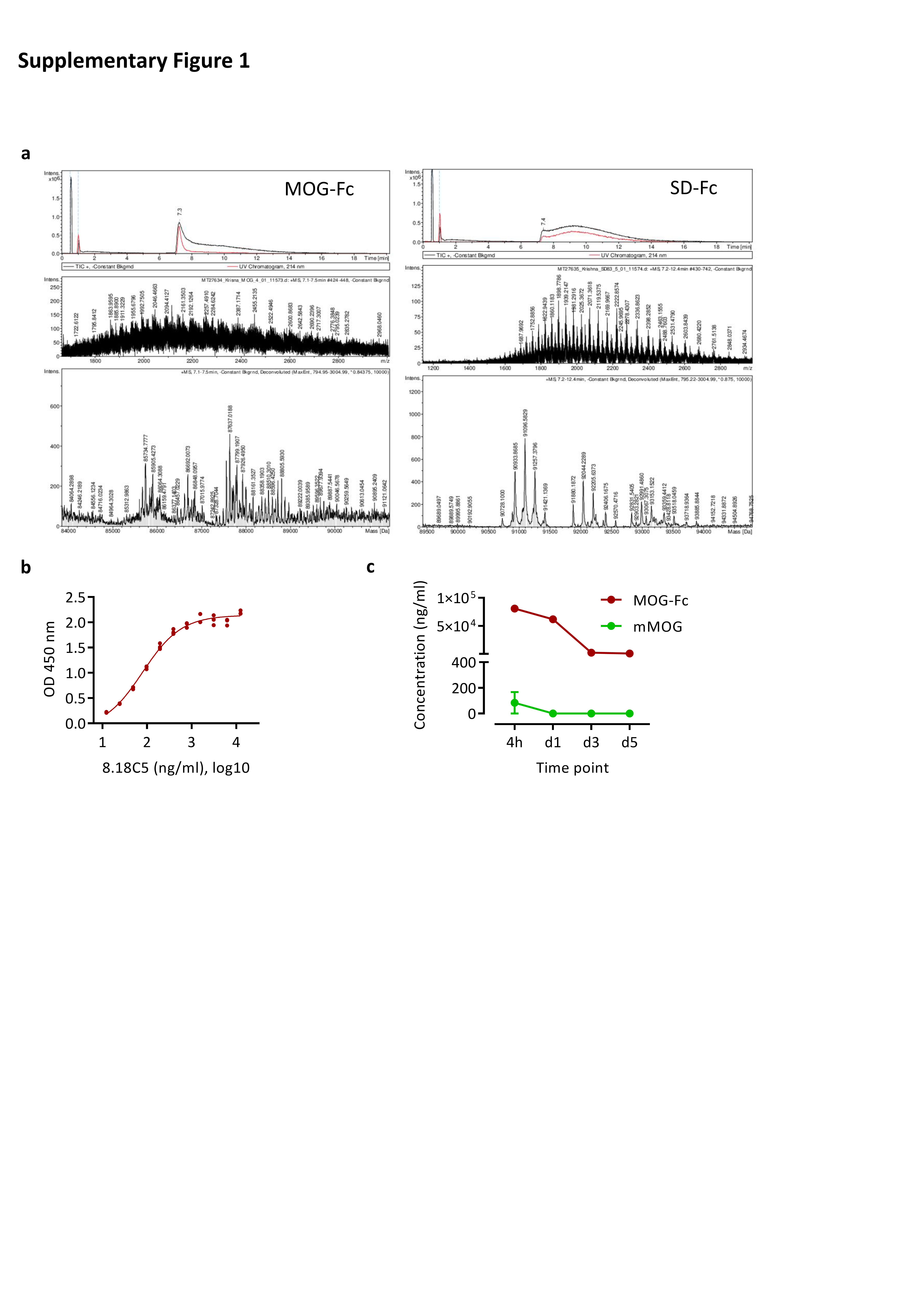

Supplement: Supplementary file 1 — Additional file 1: Figure S1. Characterization of MOG-Fc and SD-Fc proteins. a. Mass Spectrometric analysis of MOG-Fc and SD-Fc proteins. b. Dose-dependent binding of MOG-Fc to 8.18C5 (n = 3—4 replicates). anti-MOG-Fc IgG ELISA was performed and the OD at 450 nm is shown. c. Residual MOG-Fc or monomeric MOG in WT C57BL/6 mice after a single injection. 200 µg of MOG-Fc or monomeric MOG was injected into the mice (n = 4 per group) and sera were collected before injection, after 4 h, and on days 1, 3, and 5 post-injections. The serum concentration of MOG-Fc and monomeric MOG proteins is shown. [file 12974_2023_2974_MOESM1_ESM.tif]

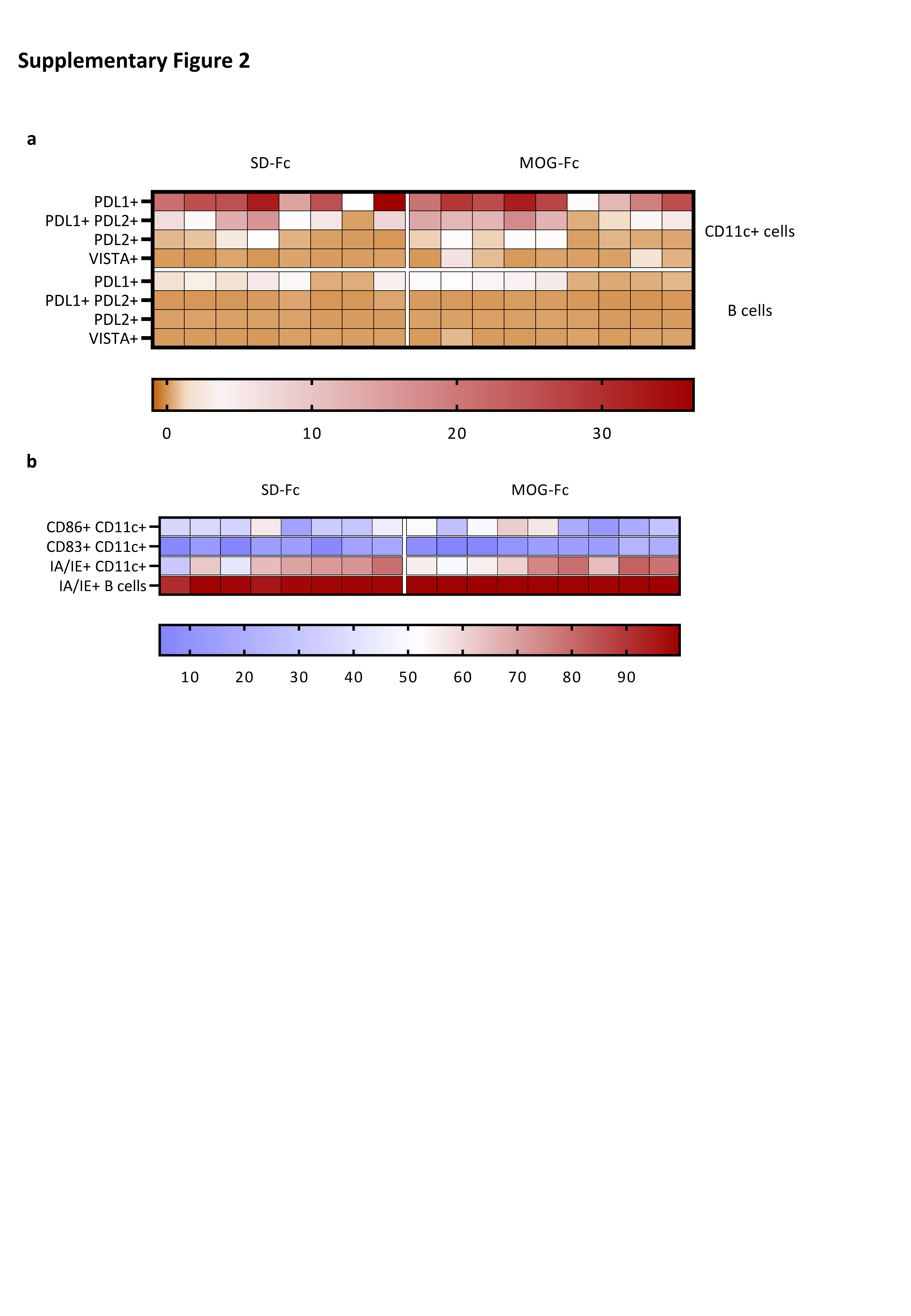

Supplement: Supplementary file 2 — Additional file 2: Figure S2. MOG-Fc does not affect non-antigen-specific APC functionality. a-b. WT C57BL/6 mice received (i.v.) OSE splenocytes along with 200 µg of MOG-Fc (n = 9) or SD-Fc (n = 8). After 3 days, lymph node cells were analyzed by flow cytometry. Data from 2 experiments are pooled. Each box in the heatmap represents one mouse. a. The frequencies of PDL1+, PDL2+, PDL1+, PDL2+, and VISTA+ populations in CD11c+ cells and B cells are represented as a heatmap. b. The frequencies of CD86+, CD83+, and IA/IE (MHC II)+ populations in CD11c+ cells and IA/IE (MHC II)+ population in B cells are represented as a heatmap. [file 12974_2023_2974_MOESM2_ESM.tif]

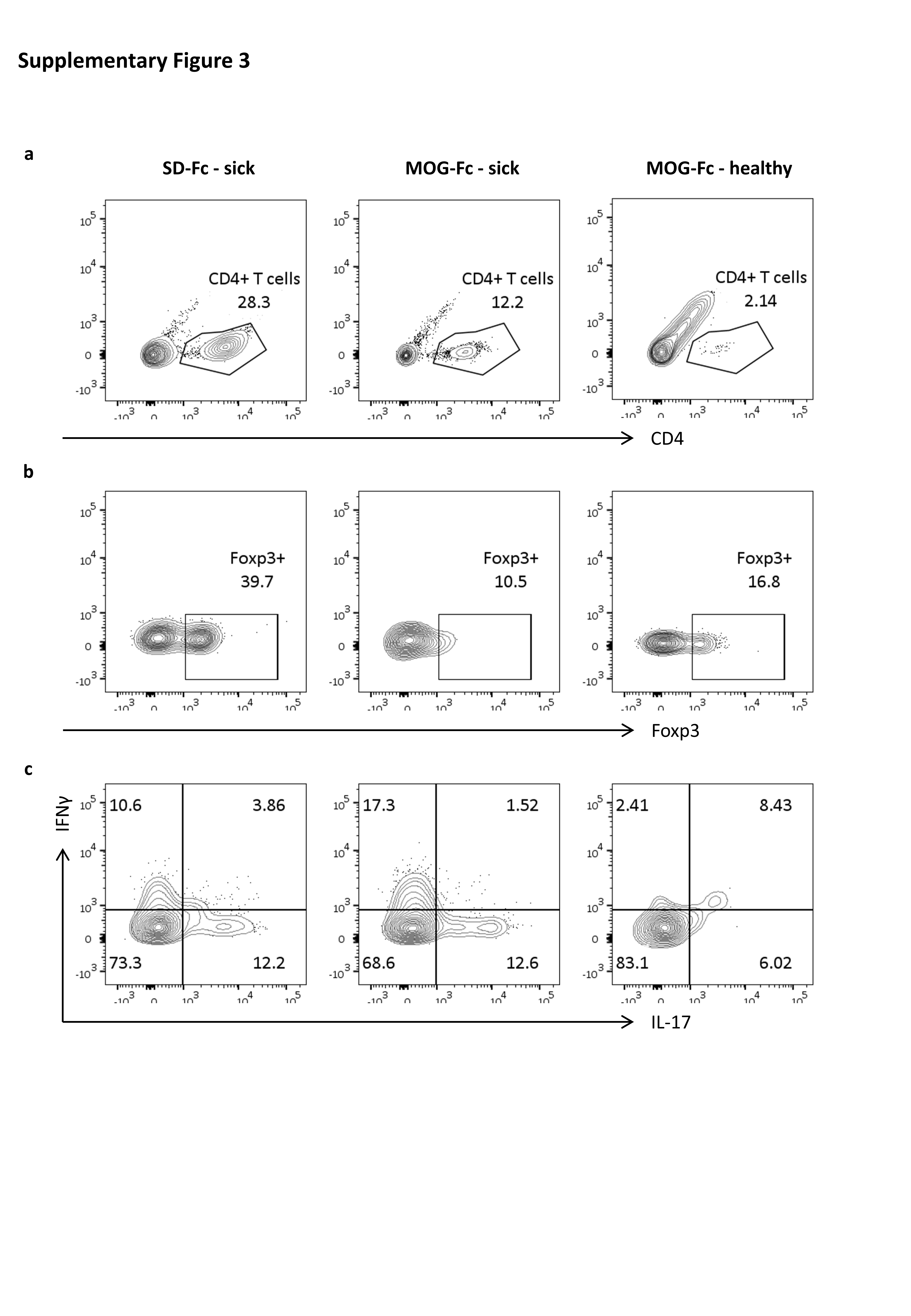

Supplement: Supplementary file 3 — Additional file 3: Figure S3. Representative flow cytometry plots for CNS infiltrates. a-c. Representative flow cytometry plots from the spinal cord of SD-Fc sick (left), MOG-Fc sick (middle), and MOG-Fc healthy (right) mice. The values on the gates show percentages (of the parent population) a. CD4 staining (gated on CD45+ cells). b. Foxp3 staining (gated on CD4+ T cells). c. IFNγ and IL-17 staining (gated on CD4+ T cells). [file 12974_2023_2974_MOESM3_ESM.tif]
