# Supplementary material for: An engineered Fc fusion protein that targets antigen-specific T cells and autoantibodies mitigates autoimmune disease
Source: J Neuroinflammation. 2023 Dec 6;20:291. doi: 10.1186/s12974-023-02974-9 (PMC10702099; doi:10.1186/s12974-023-02974-9)
Supplement: Supplementary file 4 — Additional file 4. Sequences of MOG-Fc and SD-Fc constructs. [file 12974_2023_2974_MOESM4_ESM.docx]

**Supplementary text**

**Sequence of MOG-Fc construct**


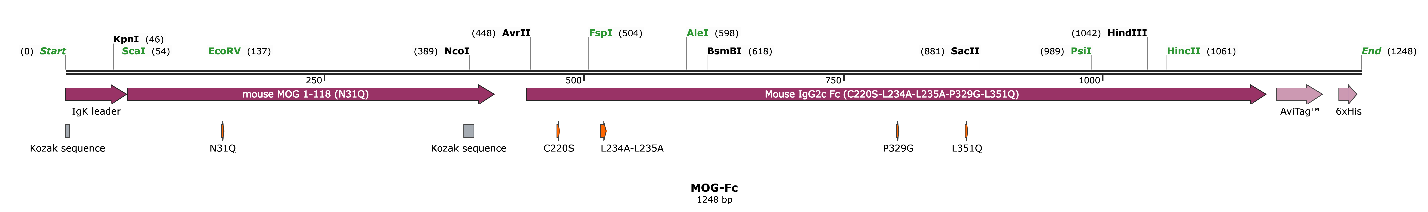


ATGGAGACAGACACACTCCTGCTATGGGTACTGCTGCTCTGGGTACCCGGAAGTACTGGAGGACAGTTCCGGGTTATCGGGCCTGGATACCCTATAAGAGCATTGGTCGGTGATGAAGCGGAACTGCCCTGTCGGATATCTCCCGGCAAACAGGCCACAGGCATGGAGGTAGGCTGGTACAGGAGTCCATTCTCCAGGGTCGTGCACTTGTACCGGAACGGTAAAGACCAGGATGCCGAACAAGCACCCGAATACAGGGGACGAACTGAGTTGTTGAAGGAGACTATATCTGAGGGGAAGGTCACACTGCGGATCCAGAATGTTCGGTTTAGCGACGAGGGTGGTTATACTTGTTTCTTTCGAGATCACTCATACCAAGAGGAAGCCGCCATGGAGCTtAAgGTGGAGGACCCAGGTGGAGGCGGGTCAGGAGGGGGTGGGTCCGAACCtAGGGTTCCTATCACACAGAATCCAaGCCCGCCGTTGAAGGAATGCCCGCCCTGCGCAGCTCCTGACGCTGCTGGTGGTCCCAGCGTTTTTATCTTCCCTCCTAAAATCAAGGACGTCTTGATGATTTCTCTGAGCCCTATGGTCACGTGCGTGGTGGTGGACGTCTCCGAGGACGACCCTGACGTCCAAATATCATGGTTCGTTAATAATGTAGAAGTACATACCGCACAGACCCAGACTCATCGCGAGGACTATAACAGTACACTTCGGGTGGTAAGCGCCTTGCCAATACAACATCAGGACTGGATGAGCGGAAAGGAGTTCAAATGTAAAGTAAATAATCGGGCTCTGGGATCCCCAATCGAAAAAACAATAAGTAAACCAAGGGGGCCGGTACGCGCCCCTCAAGTCTACGTGCAGCCACCTCCcGCGGAAGAGATGACAAAAAAGGAGTTTTCCCTTACCTGCATGATCACAGGGTTTCTTCCCGCTGAGATAGCTGTAGACTGGACCAGCAACGGCAGAACTGAGCAAAATTATAAAAACACCGCCACCGTGCTCGATAGCGACGGCTCCTATTTCATGTATTCCAAGCTTCGCGTACAGAAGTCAACCTGGGAGAGGGGTTCCCTTTTTGCCTGTTCAGTCGTACACGAGGGTTTGCATAATCACCTTACGACCAAAACTATTTCCAGAAGCCTGGGGAAAGGCAGCGGCGGCCTGAACGACATCTTCGAGGCCCAGAAGATCGAGTGGCACGAGAGCGGCGGCAGCGGCCACCACCACCACCACCACTGA

| **Feature name** | **Location** | **size (bp)** |
| --- | --- | --- |
| IgK leader | 1..60 | 60 |
| mouse MOG 1-118 (N31Q) | 61..414 | 354 |
| N31Q | 151..153 | 3 |
| Mouse IgG2c Fc (C220S-L234A-L235A-P329G-L351Q) | 445..1158 | 714 |
| C220S | 475..477 | 3 |
| L234A-L235A | 517..522 | 6 |
| P329G | 802..804 | 3 |
| L351Q | 868..870 | 3 |
| AviTag(TM) | 1168..1212 | 45 |
| 6xHis | 1228..1245 | 18 |

**Protein sequence** METDTLLLWVLLLWVPGSTGGQFRVIGPGYPIRALVGDEAELPCRISPGKQATGMEVGWYRSPFSRVVHLYRNGKDQDAEQAPEYRGRTELLKETISEGKVTLRIQNVRFSDEGGYTCFFRDHSYQEEAAMELKVEDPGGGGSGGGGSEPRVPITQNPSPPLKECPPCAAPDAAGGPSVFIFPPKIKDVLMISLSPMVTCVVVDVSEDDPDVQISWFVNNVEVHTAQTQTHREDYNSTLRVVSALPIQHQDWMSGKEFKCKVNNRALGSPIEKTISKPRGPVRAPQVYVQPPPAEEMTKKEFSLTCMITGFLPAEIAVDWTSNGRTEQNYKNTATVLDSDGSYFMYSKLRVQKSTWERGSLFACSVVHEGLHNHLTTKTISRSLGKGSGGLNDIFEAQKIEWHESGGSGHHHHHH*

**Sequence of SD-Fc construct**


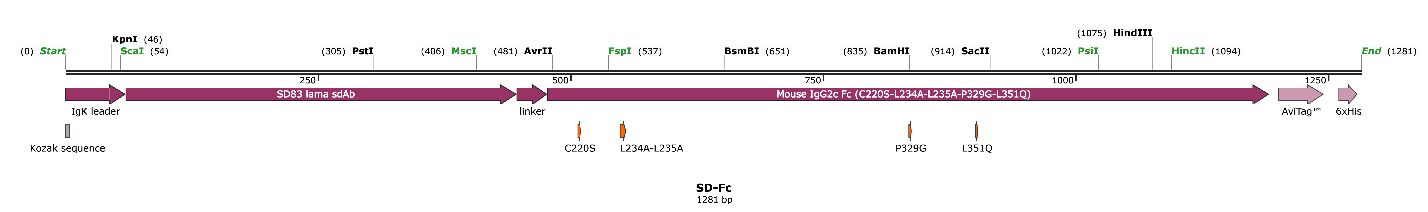


ATGGAGACAGACACACTCCTGCTATGGGTACTGCTGCTCTGGGTACCCGGAAGTACTGGAGAGGTGCAGCTGGTGGAATCTGGCGGAGGACTTGTTCAGCCTGGCGGCTCTCTGAGACTGAGCTGTGCTGCCACAGGCTTCACCCTGGAAAACAAGGCCATCGGCTGGTTCAGACAGACCCCTGGCTCTGAGAGAGAGGGCGTCCTCTGTATCAGCAAGTCTGGCAGCTGGACCTACTACACCGACAGCATGAGAGGCCGGTTCACCATCAGCAGAGACAACGCCGAGAACACCGTGTACCTGCAGATGGACAGCCTGAAGCCTGAGGACACCGCCGTGTACTACTGTGCCACAACAACAGCAGGCGGCGGACTGTGTTGGGATGGCACCACCTTTAGCAGACTGGCCAGCTCTTGGGGCCAGGGCACACAAGTGACAGTGTCTAGTGGTGGAGGCGGGTCAGGAGGGGGTGGGTCCGAACCTAGGGTTCCTATCACACAGAATCCAAGCCCGCCGTTGAAGGAATGCCCGCCCTGCGCAGCTCCTGACGCTGCTGGTGGTCCCAGCGTTTTTATCTTCCCTCCTAAAATCAAGGACGTCTTGATGATTTCTCTGAGCCCTATGGTCACGTGCGTGGTGGTGGACGTCTCCGAGGACGACCCTGACGTCCAAATATCATGGTTCGTTAATAATGTAGAAGTACATACCGCACAGACCCAGACTCATCGCGAGGACTATAACAGTACACTTCGGGTGGTAAGCGCCTTGCCAATACAACATCAGGACTGGATGAGCGGAAAGGAGTTCAAATGTAAAGTAAATAATCGGGCTCTGGGATCCCCAATCGAAAAAACAATAAGTAAACCAAGGGGGCCGGTACGCGCCCCTCAAGTCTACGTGCAGCCACCTCCCGCGGAAGAGATGACAAAAAAGGAGTTTTCCCTTACCTGCATGATCACAGGGTTTCTTCCCGCTGAGATAGCTGTAGACTGGACCAGCAACGGCAGAACTGAGCAAAATTATAAAAACACCGCCACCGTGCTCGATAGCGACGGCTCCTATTTCATGTATTCCAAGCTTCGCGTACAGAAGTCAACCTGGGAGAGGGGTTCCCTTTTTGCCTGTTCAGTCGTACACGAGGGTTTGCATAATCACCTTACGACCAAAACTATTTCCAGAAGCCTGGGGAAAGGCAGCGGCGGCCTGAACGACATCTTCGAGGCCCAGAAGATCGAGTGGCACGAGAGCGGCGGCAGCGGCCACCACCACCACCACCACTGA

| Feature name | Location | size (bp) |
| --- | --- | --- |
| IgK leader | 1..60 | 60 |
| SD83 lama sdAb | 61..447 | 387 |
| linker | 448..477 | 30 |
| Mouse IgG2c Fc (C220S-L234A-L235A-P329G-L351Q) | 478..1191 | 714 |
| C220S | 508..510 | 3 |
| L234A-L235A | 550..555 | 6 |
| P329G | 835..837 | 3 |
| L351Q | 901..903 | 3 |
| AviTag(TM) | 1201..1245 | 45 |
| 6xHis | 1261..1278 | 18 |

**Protein sequence**

METDTLLLWVLLLWVPGSTGEVQLVESGGGLVQPGGSLRLSCAATGFTLENKAIGWFRQTPGSEREGVLCISKSGSWTYYTDSMRGRFTISRDNAENTVYLQMDSLKPEDTAVYYCATTTAGGGLCWDGTTFSRLASSWGQGTQVTVSSGGGGSGGGGSEPRVPITQNPSPPLKECPPCAAPDAAGGPSVFIFPPKIKDVLMISLSPMVTCVVVDVSEDDPDVQISWFVNNVEVHTAQTQTHREDYNSTLRVVSALPIQHQDWMSGKEFKCKVNNRALGSPIEKTISKPRGPVRAPQVYVQPPPAEEMTKKEFSLTCMITGFLPAEIAVDWTSNGRTEQNYKNTATVLDSDGSYFMYSKLRVQKSTWERGSLFACSVVHEGLHNHLTTKTISRSLGKGSGGLNDIFEAQKIEWHESGGSGHHHHHH*
